# Diagnostic Testing for Differential Diagnosis in Thrombotic Microangiopathies

**DOI:** 10.4274/tjh.galenos.2019.2019.0165

**Published:** 2019-11-18

**Authors:** Gina Zini, Raimondo De Cristofaro

**Affiliations:** 1 Fondazione Policlinico Universitario A. Gemelli IRCCS - Rome, Italy; 2 Institute of Hematology, Università Cattolica del S. Cuore, Rome, Italy; 3 Institute of Internal Medicine and Geriatrics, Università Cattolica del S. Cuore, Rome, Italy

**Keywords:** Microangiopathic hemolytic anemia, Thrombotic microangiopathies, Anemia

## Abstract

Thrombotic microangiopathies (TMAs) are multiple disease entities with different etiopathogeneses, characterized by thrombocytopenia, microangiopathic hemolytic anemia (MAHA) with schistocytosis, variable symptoms including fever, and multi-organ failure such as mild renal impairment and neurological deficits. The two paradigms of TMAs are represented on one hand by acquired thrombotic thrombocytopenic purpura (TTP) and on the other by hemolytic uremic syndrome (HUS). The differential diagnosis between these two paradigmatic forms of TMA is based on the presence of either frank renal failure in HUS or a severe deficiency (<10%) of the zinc-protease ADAMTS13 (a disintegrin and metalloproteinase with a thrombospondin type 1 motif, member 13) in TTP. ADAMTS13 is an enzyme involved in the proteolytic processing of von Willebrand factor (vWF), and its deficiency results in formation of high-molecular-weight vWF-rich microthrombi in the environment of the microvasculature. The presence of these ultra-large vWF multimers in the microcirculation can recruit platelets, promoting multi-organ ischemic lesions. The presence of ADAMTS13 activity at >10% could rule out the presence of a TTP form. However, it is often difficult to differentiate either a TTP or HUS clinical scenario presenting with typical symptoms of TMA. There are in fact several additional diagnoses that should be considered in patients with ADAMTS13 activity of >10%. Widespread inflammation with endothelial damage and adverse reactions to drugs play a central role in the pathogenesis of several forms of TMA, and in these cases, the differential diagnosis should be directed at the underlying disease. Hence, a correct etiologic diagnosis of TMA should involve a critical illness, cancer-associated TMA, drug-induced TMA, and hematopoietic transplant-associated TMA. A complete assessment of all the possible etiologies for TMA symptoms, including acquired or congenital TTP, will allow for a more accurate diagnosis and application of a more appropriate treatment.

## Abstract (tr)

Trombotik mikroanjiyopatiler (TMA), farklı etiyopatogenezleri olan; trombositopeni, şistositlerin eşlik ettiği mikroanjiyopatik hemolitik anemi (MAHA), ateş, hafif böbrek yetmezliği ve nörolojik defisitler gibi çoklu organ tutulumlarıyla karakterize bir hastalıklar grubudur. TMA’ların iki paradigması bir yandan edinsel trombotik trombositopenik purpura (TTP) ve diğer yandan hemolitik üremik sendrom (HUS) ile temsil edilir. TMA’nın bu iki paradigmatik formu arasındaki ayırıcı tanı, HUS’de belirgin böbrek yetmezliği veya TTP’de çinko-proteaz ADAMTS13’ün (bir disintegrin ve metalloproteinaz trombospondin tip 1, üye 3) ciddi eksikliğinin (<%10) varlığına dayanmaktadır. ADAMTS13, von Willebrand faktörünün (vWF) proteolitik işleminde yer alan bir enzimdir ve eksikliği, mikrovasküler ortamda yüksek molekül ağırlıklı vWF bakımından zengin mikrotrombüs oluşumuna yol açar. Bu ultra-büyük vWF multimerlerinin mikro dolaşımdaki varlığı, trombositleri toplayarak çok organlı iskemik lezyonları teşvik eder. ADAMTS13 aktivitesinin %10’dan büyük oluşu, bir TTP formunun varlığını dışlatabilir. Bununla birlikte, tipik TMA semptomları gösteren bir TTP veya HUS klinik senaryosunu ayırt etmek genellikle zordur. Aslında ADAMTS13 aktivitesi %10’dan büyük olan hastalarda göz önünde bulundurulması gereken birkaç ek tanı vardır. Endotel hasarı ve ilaçlara verilen yan etkilerle birlikte görülen yaygın yangı, birçok TMA formunun patogenezinde merkezi bir rol oynamaktadır ve bu durumlarda, ayırıcı tanı altta yatan hastalığa yönlendirilmelidir. Bu nedenle, TMA’nın doğru bir etiyolojik tanısı, kritik bir hastalık, kansere bağlı TMA, ilaca bağlı TMA ve hematopoetik transplant ile ilişkili TMA’yı içermelidir. Edinilmiş veya konjenital TTP dahil olmak üzere TMA semptomları için olası tüm etiyolojilerin tam bir değerlendirmesi, daha uygun bir tedavinin daha doğru bir şekilde teşhis edilmesine ve uygulanmasına olanak sağlayacaktır.

## Introduction

The name thrombotic microangiopathy (TMA) refers to rare multisystem diseases characterized by damage of endothelial walls of arterioles and capillaries, which leads to massive occlusion and formation of platelet-rich thrombi and microangiopathic hemolytic anemia (MAHA). By definition, TMA indicates neither a specific diagnosis nor a specific etiology; it is just a pathologic diagnosis made by tissue biopsy [1,2]. TMAs are medical emergencies requiring rapid diagnosis and appropriate treatment.

The term MAHA refers to nonimmune hemolytic anemia caused by red blood cell (RBC) intravascular fragmentation. This is combined with:

-  schistocytosis, with a confidence threshold of 1% in peripheral blood to support a clinical diagnosis of TMA [3,4];

-  consumption thrombocytopenia with platelets of <150x10^9^ or a decrease from baseline of >25%.

-  negative direct antiglobulin test (DAT);

- indirect indicators of hemolysis, such as increased plasma lactate dehydrogenase (LDH), and/or decreased hemoglobin and/or haptoglobin;

-  fever and organ involvement, including renal impairment and/or neurological, gastrointestinal, cardiovascular, pulmonary, or visual symptoms.

Not all cases of MAHA are caused by a TMA, but all TMAs cause MAHA and thrombocytopenia.

## History

Moschcowitz in 1924 described for the first time a case of abrupt onset and progression of petechial bleeding, pallor, fever, paralysis, hematuria, and coma [5], with disseminated microvascular hyaline thrombi in arterioles and capillaries. In 1947 Singer et al. [6] first introduced the term “thrombotic thrombocytopenic purpura” (TTP). The name TMA was introduced by Symmers in 1952 to describe the vascular lesions observed in TTP [7]. In 1955 Gasser et al. [8] described the symptoms of a child with thrombocytopenia, hemolytic anemia, and renal failure with bilateral diffuse cortical necrosis: this was called hemolytic uremic syndrome (HUS). In 1982 Moake et al. [9] suggested a defective processing of ultra-large von Willebrand factor (vWF) multimers produced by endothelial cells. In 1983, Karmali et al. [10] associated HUS with infections with *Escherichia coli* producing Shiga toxin (ST). According to Furlan et al. [11], increased proteolytic cleavage of vWF is observed in a number of cases with type 2A von Willebrand disease. Large vWF multimers, which are hemostatically active, are degraded to form smaller and less active molecules. In particular, the peptide bond between 842Tyr and 843Met is cleaved in the polypeptide subunits of vWF. The increased frequency of platelet thrombosis in TTP patients is related to a deficiency of such proteolytic activity [12,13]. The key vWF-cleaving protease, on the basis of partial amino acid sequencing, was a large zinc-containing metalloprotease, identified as “a disintegrin and metalloproteinase with thrombospondin type 1”, member 13 (ADAMTS13) of the ADAMTS protease family [14,15].

## Epidemiology and Pathogenesis of TMA

TMAs are rare diseases: five to ten cases/year per million cases of TTP are acquired, with a male:female ratio of 1:2 and a peak of incidence during the 4^th^ decade of life. Hereditary TTP represents one or fewer cases/year per million [19,20].

The most prominent diagnoses associated with TMA are thrombotic TTP and HUS. They usually occur, respectively, in adults and in children. As discussed below, their pathogenesis is different: TTP results from a severe ADAMTS13 deficiency, which can be caused by circulating autoantibodies or ADAMTS13 mutations, while HUS is correlated to infection with ST-producing bacteria or gene mutations causing an excess of activation of the alternative pathway [16]. According to recent observations in TTP/HUS registries, emerging features of these disorders are the diagnostic value of ADAMTS13 measurement, efficacy of plasma exchange (PEX), and frequency of relapses after remission [17,18].

Many different disorders can cause TMA (i.e. secondary TMA; see below).

Other clinical TMA presentations are:

-   HELLP syndrome (hemolysis, elevated liver enzymes, low platelet count), which is observed in a proportion of 0.5%-0.9% of pregnancies, as well as in 10%-20% of severe preeclampsia cases [21];

-   catastrophic antiphospholipid syndrome, which is rarely observed patients with acute multi-organ thrombosis (less than 1%);

-   malignant hypertension, in about 2.6 cases/year per 100,000 cases with a higher incidence among people of African descent;

-   cancer: about 5% of patients with disseminated malignancy;

-   transplant-associated TMA following a) non-renal solid organ transplantation (incidence 5%, 4.0% in liver, 2.3% in lungs) [22,23], b) renal transplantation, with 5.6/1000/year with a 50% mortality rate at three years [24], and c) hematopoietic progenitor cell transplantation, with variable ranges from 0% to 74% and median incidence of 7.9% [2,25].

Finally, TMAs are also part of the pathology of disseminated intravascular coagulation (DIC), in which it results from the deposition of fibrin or platelets within the microvasculature [26], and scleroderma renal crisis [27]. In Table 1 the TMAs are listed according to cause.

This review mainly deals with diagnostic aspects of MAHA and TMAs. A number of clinical problems await solutions in TMA, such as the positioning of rituximab in the treatment sequence of primary TTP, management of ST-producing *Escherichia coli*-HUS complicated by encephalopathy, the efficacy and long-term safety of eculizumab in atypical HUS, and elucidation of the pathogenesis of secondary TMA [28,29,30].

## Clinical Forms of TMA

TTP is a clinical emergency with a mortality rate of up to 90% if not promptly treated [31]. African-Caribbean ancestry [32] and obesity [33] are risk factors. It is caused by a lack or deficiency of ADAMTS13. In normal individuals, endothelial cells produce vWF multimers from the Weibel-Palade bodies and the metalloprotease enzyme ADAMTS13 cleaves the unusually large multimers, avoiding platelet adhesion [34]. When the vWF multimers are not cleaved, platelets adhere and the endothelial layers of small vessels are damaged, causing platelet aggregation and fibrin deposition in microcirculation. Infections, drugs, and pregnancy/delivery [35,36] may act as triggers in predisposed individuals. ADAMTS13 activity may be absent or highly inhibited by circulating autoantibodies, which represent the most frequent cause of acquired TTP. Up to 75% of patients in the acute phase show the presence of IgG immunoglobulins with anti-ADAMTS13 activity, which inhibit its proteolytic activity towards vWF. Such autoantibodies circulate in the form of immuno-complexes (IC) and are the cause of the deficiency of ADAMTS13. In 20%-25% of patients anti-ADAMTS13 autoantibodies are not detectable, so that the mechanisms that underlie ADAMTS13 deficiency are not fully clarified. Less than 5% of TTP cases are due to *ADAMTS13* gene mutation (congenital TTP, Upshaw-Schulman syndrome (USS), an autosomal recessive disease presenting with early onset in childhood) [37,38]. More than 150 different *ADAMTS13 *gene mutations have been described to date: 70% of these mutations are missense, while the remaining 30% are truncating [37]. In the suspicion of a congenital form of HUS, the ADAMTS13 level should be evaluated by measuring both its activity with a fluorogenic assay [39] and its antigen level to differentiate between type 1 (both activity and antigen decreased) and type 2 deficiency (severe activity defect associated with subnormal antigen level).

ST-mediated HUS is associated with the microbiological finding of *Escherichia coli*, mainly O157:H7 and O104:H4 serotypes, and/or* Shigella dysenteriae *type 1 infection: the production of the ST leads to endothelial and glomerular damage with an acute clinical picture. It is usually caused by food, with a seasonal distribution with a summer peak, and it represents the main cause of acute renal impairment in children less than 3 years old. Enterohemorrhagic diarrhea self-resolves in most cases, but in 5%-7% of them, HUS develops a few days afterwards. ST, a pentamer of B subunits, causes endothelial cell damage through binding to a globotriaosylceramide receptor expressed on the membrane of endothelial cells: after internalization by endocytosis, ST inhibits protein synthesis, causing cell apoptosis and death [40] and exposure of the extracellular matrix with platelet aggregation, fibrin deposition, and mechanical hemolysis. The kidneys, gastrointestinal tract, and central nervous system (CNS) are the key target organs. ST-mediated HUS, which can be as severe as acute HUS, reaches a mortality rate of up to 5% [41].

Complement-mediated TMA presents with thrombocytopenia, mechanical hemolysis, and acute renal failure, with severe arterial hypertension and ischemic damage due to activation and/or abnormal regulation of the alternative pathway of the complement system on cell surfaces: mutations in C3 and factor B; autoantibodies against factor H interfering with regulation; disturbed recognition by factor H, factor I, or CD46 of C3b; and disturbed recognition by factor H of self-cell surface molecules, such as sialic acid or glycosaminoglycans [42]. About 20% of cases show a subclinical onset, with slow disease progression [43].

Coagulation-mediated TMA is caused by mutations of genes encoding for thrombomodulin (*THBD*), plasminogen (*PLGx*), and diacylglycerol kinase epsilon (*DGKE*), inducing upregulation of prothrombotic factors [44,45].

Metabolism-mediated TMA, usually seen in infants, is caused by mutations in different genes that cause methylmalonic and aciduria homocystinuria type C (*MMACHC*) [46].

Drug-mediated TMA [47] can be caused by:

-  immune-mediated mechanisms with antibodies formation (quinine) [48].

-  dose-dependent/toxicity mechanisms (cyclosporine, tacrolimus, clopidogrel, interferon, vascular endothelial growth factor inhibitor, mitomycin C).

-  induction of drug-independent antibodies (ticlopidine).

New observations are not rare, such as TMA associated with the intravenous injection of adulterated Opana ER tablets [49].

Secondary TMAs are caused by different coexisting disorders, such as systemic infections [50]. In particular, infections due to *Streptococcus pneumoniae* and influenza viruses are considered true etiological factors, instead of simple triggers, of TMA. Cancer [51], transplantation of bone marrow or solid organs [52], autoimmune disease [53], pregnancy [54], cytotoxic drugs, DIC, severe deficiency of vitamin B_12_ [55], and pancreatitis can be responsible for the development of secondary TMA. A common feature of the above-mentioned conditions is the generation of direct cell damage, with general activation of the complement system or enhanced activation of the complement on cell membranes [42].

## Diagnostic Tests

**Figure 1:**
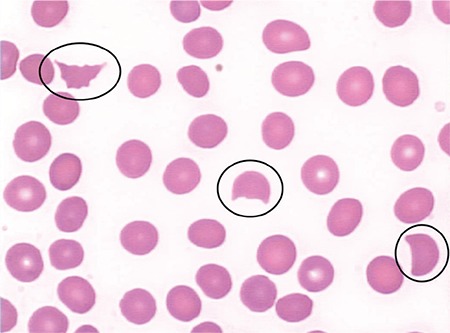
Schistocytes should be evaluated on smears at medium microscope magnification.

**Figure 2:**
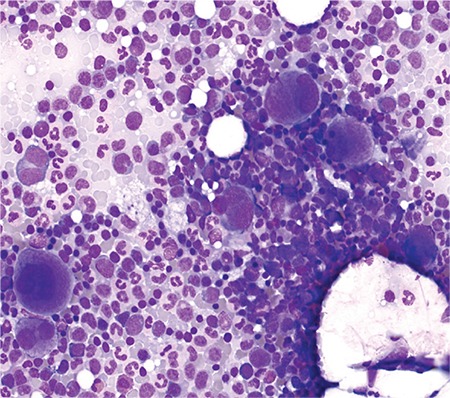
Increased megakaryocytes in bone marrow associated with thrombocytopenia testify to the presence of peripheral platelet consumption.

Almost all cases of TMA are associated with MAHA. It is extremely important to exclude at a clinical level any possible cause of MAHA alternative to TMA. In particular, occasionally patients with paroxysmal nocturnal hemoglobinuria, intravascular and/or heart devices, heparin-induced thrombocytopenia, and systemic disorders such as systemic infections can present with MAHA in association with or without TMA. The main causes of secondary TMAs were mentioned above; the patient’s history and physical examination are fundamental steps for the most appropriate diagnostic pathway. Diagnosis of MAHA is confirmed by negativity of DAT, increased LDH, and/or decreased haptoglobin. Organ involvement should be investigated. Complete blood count in MAHA shows normocytic anemia, reticulocytosis, and severe thrombocytopenia, while in the peripheral blood smear schistocytes, microspherocytes, and polychromatophilic RBCs, identifiable as immature reticulocytes by vital stains, are detected. Schistocytes are fragmented red cells appearing in a variety of shapes: rectangular, crescent, or helmet-shaped. Traditionally they are identified and counted by microscopic observation by trained laboratory scientists, with a large margin of error [3]. In TMA, RBCs are physically sheared by fibrin networks in the peripheral circulation: the appearance of schistocytes may be one of the earliest signs of TMA and its detection and quantitation are of primary importance. In 2012 the International Council for Standardization in Haematology published speciﬁc recommendations to standardize schistocyte identiﬁcation, enumeration, and reporting [3], including morphological criteria for the identification of specific schistocyte types. Reference values are ≤0.1% in adults, 0.3%-1.9% in newborns, and ≤5.5% in preterms. Schistocytes should be evaluated on smears at medium microscope magniﬁcation as a percentage after counting at least 1000 red blood cells (Figure 1). Schistocyte count has deﬁnite clinical value for diagnosis of TMA in the absence of additional severe red cell shape abnormalities, with a conﬁdence threshold value of 1%. Fragmented RBC enumeration by automated counters is a complement to microscopy, providing rapid results with high predictive value for negative samples [3,4]. Increased megakaryocytes in bone marrow (Figure 2), usually with left shift, associated with thrombocytopenia testify to the presence of peripheral platelet consumption. Bone marrow aspiration is not mandatory but can facilitate the differential diagnosis (versus promyelocytic leukemias with DIC or other hypoplastic/aplastic marrow diseases, including hemophagocytic syndrome).

Once primary TMA is confirmed, the type should be determined to provide the patient with the specific treatment: PEX in TTP and eculizumab in complement-mediated TMA. The patient’s sample for assay of ADAMTS13 functional levels should be investigated.

ADAMTS13 activity measurements (degradation of a vWF substrate) are currently based on different methods [56]: fluorescence resonance energy transfer (FRET) [57], chromogenic enzyme-linked immunosorbent assay (ELISA) [58], mass spectrometry [59], and simplified methods based on coagulation analyzers [60].

Results of ADAMTS13 measurements are reported as a percentage of ADAMTS13 activity in pools of plasma from healthy donors, with a threshold of <10%. It is possible, however, in the opinion of these authors, that a lower threshold should be considered, given the increased sensitivity of new-generation methodologies. An international World Health Organization standard plasma method for the measurement of ADAMTS13 has recently become available [61]. DNA testing for ADAMTS13 genes has also been developed [62].

Clinical interpretation is fundamental because of possible false low results due to hemolysis or increased bilirubin, especially in FRETS-based assays. Moreover, unfortunately results of the diagnostic tests are not immediately available, while patients with acute MAHA and thrombocytopenia usually require immediate treatment. In this scenario the PLASMIC score [63] does represent immediate help in calculating the diagnostic probability of TTP, evaluating very simple parameters/information. One point is assigned to each of the following:

i) platelet count of <30x10^9^/L;

ii) plasma or serum indirect bilirubin >2 mg/dL, *or* reticulocyte count >2.5%, *or* undetectable plasma haptoglobin,

iii)  absence of active cancer,

iv) absence of solid organ or stem cell transplant in the medical history,

v) mean corpuscular volume (MCV) of <90 fL,

vi) international normalized ratio (INR) <1.5,

vii) plasma or serum creatinine <2.0 mg/L.

**Figure 3:**
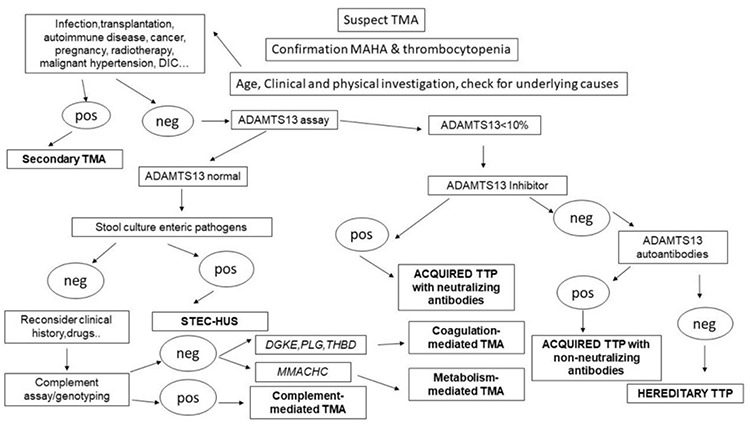
Algorithm for differential laboratory diagnosis in patients with clinical suspicion of thrombotic microangiopathie.

The PLASMIC risk score for severe ADAMTS13 deficiency can be low (<5), intermediate (5), or high (>5). ST-HUS acute onset is characterized by abdominal pain, associated with vomiting and bloody diarrhea, which can anticipate by several days other clinical and laboratory signs of MAHA associated with thrombocytopenia. Stool cultures for enteric pathogens do confirm the correct diagnosis. In complement-mediated TMA, symptoms are less typical, more insidious, and generic (acute renal failure, edema); up to 20% of cases present with multi-organ failure (CNS, cardiac, pulmonary, intestinal). It is reported as familial and sporadic, presenting in up to 80% of children and 50% of adults [64,65]. Quantitative, genetic, and functional complement assessment will lead to the diagnosis, and while waiting for lab test results it is mandatory to start treatment with PEX, moving to anti-complement therapy after obtaining the results. In drug-mediated TMA, supportive therapy and drug discontinuation are indicated, while in metabolism-mediated TMA and coagulation-mediated TMA the role of molecular testing is fundamental. Figure 3 displays an algorithm for differential laboratory diagnosis in patients with clinical suspicion of TMA.

## Conclusion

The differential diagnosis of TTP, HUS forms, and TMA from other etiologies can be challenging. Diagnosis has to be primarily based on clinical history (underlying disease, medications). In intensive care patients, TMA is more probably associated with the underlying illnesses. In patients presenting with TMA signs, clinical antecedents of metastatic malignancy, hypertension, polychemotherapy or immunosuppressive treatment, HELLP syndrome, or allogeneic stem cell transplant should be considered as possible causes for the TMA presentation. In the great majority of such patients, a serum level of ADAMTS13 activity lower than 10% is a useful element for the differential diagnosis. Finally, not infrequently diagnostic assessment has to be extended after treatment and recovery of patients, especially when biochemical and molecular biology studies, including mutation analysis of complement factors, may add useful elements.

## Figures and Tables

**Table 1:**
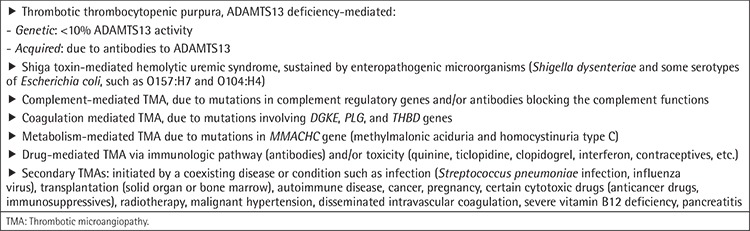
Thrombotic microangiopathies listed according to causes.
